# Seasonality in carriage of extended-spectrum *β*-lactamase-producing *Escherichia coli* and *Klebsiella pneumoniae* in the general population: a pooled analysis of nationwide cross-sectional studies

**DOI:** 10.1017/S0950268820000539

**Published:** 2020-02-21

**Authors:** C. C. H. Wielders, E. Van Duijkeren, G. Van Den Bunt, A. P. Meijs, C. M. Dierikx, M. J. M. Bonten, W. Van Pelt, E. Franz, S. C. De Greeff

**Affiliations:** 1 Centre for Infectious Disease Control (CIb), National Institute for Public Health and the Environment (RIVM), Bilthoven, The Netherlands; 2 Julius Center for Health Sciences and Primary Care, University Medical Center Utrecht, Utrecht, The Netherlands; 3 Department of Medical Microbiology, University Medical Center Utrecht, Utrecht, The Netherlands

**Keywords:** Antibiotic resistance, Enterobacteriaceae, prevalence, seasonality

## Abstract

Infections due to extended-spectrum *β*-lactamase-producing Enterobacteriaceae (ESBL-E) are often preceded by asymptomatic carriage. Higher incidences in enteric infectious diseases during summer have been reported. Here, we assessed whether the presence of seasonality in intestinal ESBL-*Escherichia coli*/*Klebsiella pneumoniae* (ESBL-E/K) carriage in the general Dutch population exists. From 2014 to 2017, the faecal carriage of ESBL-E/K in healthy individuals was determined in three cross-sectional studies in the Netherlands, including 5985 subjects. Results were pooled to identify seasonal trends in prevalence (by month of sampling). Multivariate logistic regression analysis was used to calculate pooled odds ratios and 95% confidence intervals. Results were adjusted for age, sex, antibiotic use and travel. Overall prevalence of ESBL-E/K carriage was 4.3% (*n* = 260 ESBL-E/K-positive), with differences between months ranging from 2.6% to 7.4%. Compared to January, the monthly prevalence of ESBL-E carriage was highest in August (OR 1.88, 95% CI 1.02–3.49) and September (OR 2.25, 95% CI 1.30–3.89). The observed monthly differences in ESBL-E/K carriage rates suggest that there is seasonal variation in exposure to ESBL-E/K other than due to travelling and antibiotic use. This should be taken into account in designing future ESBL-E prevalence studies in temperate regions.

## Text short report

Enterobacteriaceae that produce extended-spectrum *β*-lactamases (ESBLs) are an important reason for therapy failure with *β*-lactam antibiotics [1]. Infections due to ESBL-producing Enterobacteriaceae (ESBL-E) are often preceded by asymptomatic carriage [2]. Besides human-to-human transmission, potential routes of transmission of ESBL-E to humans are the food chain, direct contact with animals or indirectly via the environment [3]. The prevalence of the faecal carriage of ESBL-E in the general population in the Netherlands is approximately 5% [4–6].

With enteric zoonotic diseases (including Salmonellosis, Campylobacteriosis and infections with Shiga toxin-producing *Escherichia coli*), seasonal variation is ubiquitous in temperate regions, with increased human disease incidence during warmer months [7]. In general, this increased incidence is following the increased pathogen prevalence and shedding from animal reservoirs during warmer months [7]. Indeed, during warmer months, often an increase in the environmental presence of *E. coli* is observed [8]. On the other hand, higher temperature is associated with reduced *E. coli* survival in water and soil, which is probably related to the increased competition for nutrients following the increased activity of environmental bacteria at higher temperatures [9].

Seasonality in healthcare-associated infections, mainly bloodstream infections (BSIs), caused by Gram-negative bacteria including *E. coli* and *Klebsiella pneumoniae* has been shown in several studies with higher BSI incidence rates with temperature increase, although for *K. pneumonia* this association was not always observed [10]. More recent studies showed that mainly community-acquired *E. coli* BSIs, in contrast to hospital-acquired BSIs, showed higher rates during warmer months [11, 12]. However, ESBL-E was not assessed in these studies. A recent ecological study on antibiotic resistance in *E. coli*, *K. pneumoniae* and *Staphylococcus aureus* using antibiotic resistance pattern data from hospitals, laboratories and surveillance units in the USA showed that the prevalence of antibiotic resistance increases with local temperature (increase in average minimum temperature) [13].

So far, seasonal variation in asymptomatic ESBL-E carriage in the general population has not been investigated. Two studies investigated the seasonal trends of ESBL-carriage in patients. One study by Mesa *et al*. on the faecal carriage of ESBL-E in a limited number of patients attending an emergency room of two hospitals in Barcelona and persons involved in food-borne outbreaks showed that there was a temporal distribution in ESBL-E carriage with a decrease in the prevalence in May and an increase in July [14]. However, data were solely descriptive without adjustment for confounders such as previous travel or antibiotic use, and sampling was not performed year-round. A second study by Kaier *et al*. assessed the incidence of carriage (including both colonised persons and persons with infections) of ESBL-producing *E. coli* and *K. pneumoniae* (ESBL-E/K) in hospitalised patients in two German university hospitals by using routine laboratory surveillance data. They found an increasing trend in the incidence of ESBL-E/K carriage during summer compared to winter [15].

It is important to know whether seasonality (or monthly variations) is present in the asymptomatic carriage of ESBL-E in order to design future prevalence studies more appropriately by decreasing the chance of finding factors associated with ESBL-E carriage which is due to a difference in exposure (i.e. moment of sampling). The aim of the present study is to assess whether there is a seasonal difference in the carriage of ESBL-E/K in the general Dutch population. The outcomes of this pooled analysis may also help to better understand the variations in ESBL-E/K carriage rates.

From 2014 to 2017, the faecal carriage of ESBL-producing *E. coli* and *K. pneumoniae* was determined in three different large cross-sectional studies in the Netherlands in the selections of predominantly healthy individuals in the population at large: the Livestock Farming and Neighbouring Residents' Health Study (VGO) [5], the ESBL-population study (ESBLAT) [6] and a study on ESBL-producing bacteria among vegetarians and non-vegetarians (Vega Study) [16]. The VGO study was performed from February 2012 to May 2014 among the general population aged 18–70 years old living in a livestock-dense area in the south of the Netherlands, and after consent to participate in further studies, ESBL-E/K faecal carriage was assessed between February 2014 and May 2015 [5]. In the ESBLAT study, a random sample of 2000 persons was invited every month from the Dutch Personal Records Database to submit faecal samples between October 2014 and February 2017. In the Vega Study, vegetarians and persons eating meat were actively contacted to submit faecal samples in order to study ESBL-E/K carriage between November 2015 and March 2017. More characteristics of the three studies are described in Table 1 and details of the respective studies and laboratory methods can be found in the cited references [5, 6, 16]. All individual level data of the three studies were combined into a new dataset in order to perform a pooled analysis. Pooling was carried out since the participants in all three studies were sampled from the general population, were aged ≥18 years and similar laboratory techniques to identify ESBL-E/K carriers were performed. Participants were only included in the analysis when (i) aged ≥18 years, (ii) data on antibiotic use in the last 6 months were available and (iii) data on travelling in the last 6–12 months were available. Two participants of the ESBLAT study carrying ESBL-producing *Enterobacter cloacae* complex were excluded from our analyses. Data on antibiotic use and travel were collected via self-reported questionnaires in all three studies, except for antibiotic use in the VGO study. In the VGO study, antibiotic use was derived from general practitioner (GP) electronic medical records and data were only included when the GP registered prescriptions for ≥46 weeks during the calendar year and if the patient was registered at the particular GP for at least three-quarters of the year [5]. Data on travelling in the last 6 months were available for participants from the Vega Study and for the last 12 months for the VGO and ESBLAT studies (Table 1). Of note, vegans and vegetarians participating in the Vega Study were excluded from the pooled analysis, since including them would result in a non-representative sample of the adult general Dutch population, as around 95% of the population eats meat. We therefore only included participants from the Vega Study who reported to eat meat at least once per week. However, our analysis still included vegetarians that participated in the VGO and ESBLAT studies.

**Table 1.** Characteristics of the cross-sectional studies performed in the Netherlands (2014–2017) and included in the pooled analysis

Study	Time period	Population original study	Travel	Antibiotic use	Number of participants in original study	Number of ESBL-E-positive participants in original study and prevalence	Number and percentage of participants included in pooled analysis	Number of ESBL-E/K-positive participants included in pooled analysis and prevalence
					*n*	*n* (%)	*n* (%)	*n* (%)
Livestock Farming and Neighbouring Residents' Health Study (VGO) [5]	February 2012–May 2014 recruitment, February 2014–May 2015 participation	General population living in a livestock-dense area, selected via a two-step procedure: first recruited via their general practitioner (GP) through a short questionnaire survey, then one person per household aged 18–70 years living in a municipality with <30 000 inhabitants in the eastern part of the province of Noord-Brabant or northern part of the province of Limburg and living within 10 km of one of the 12 established research centres	During last 12 months	During last 6 months	2432	109 (4.5)	2012 (82.7)^a^	80 (4.0)
ESBL-ATtribution study (ESBLAT) [6]	October 2014–November 2016 recruitment, October 2014–February 2017 participation	Random sample of the Dutch population by a monthly sample from the Dutch Personal Records Database, all ages	During last 12 months	During last 6 months	4177	186 (4.5)	3557 (85.2)^b^	158 (4.4)
Study on ESBL-producing bacteria among vegetarians and non-vegetarians (Vega Study) [16]	November 2015–March 2017 recruitment, November 2015–April 2017 participation	Vegans, vegetarians, pescatarians and people who do eat meat recruited from VegFest 2015 (a trade fair for vegans), via messages on Twitter and Facebook and news items in the media, age ⩾18 years	During last 6 months	During last 6 months	1634	114 (7.0)	414 (25.3)^c^	22 (5.3)
Total					8636	385 (4.5)	5983 (69.3)	260 (4.3)

a Exclusion because of missing data on antibiotic use from general practitioners (*n* = 392), missing data on travel (*n* = 20) or both antibiotic use and travel data missing (*n* = 8).

b Exclusion because of age <18 years (*n* = 574), unknown date of sampling (*n* = 2), unknown age (*n* = 1), ESBL-producing *Enterobacter cloacae* complex (*n* = 2), missing data on antibiotic use (*n* = 20), missing data on travel (*n* = 19) or both antibiotic use and travel data missing (*n* = 2).

c Exclusion because of not eating meat (*n* = 1177), eating meat less than once per week (*n* = 42) or missing data on travel (*n* = 1).

Results from the three studies were pooled to study the presence of seasonal trends in prevalence throughout the year (by month of sampling). The trend in monthly prevalence was tested using the Mantel–Haenszel *χ*^2^ test. A *P*-value <0.05 was considered statistically significant. Univariate and multiple logistic regression analysis was performed and pooled odds ratios (ORs) and 95% confidence intervals (95% CIs) were calculated. All variables available were included into the multivariate analysis since these were either general characteristics (age, sex), known risk factors for ESBL-E/K carriage (antibiotic use during the last 6 months and travel during the last 6–12 months) or our variable of interest (month of sampling). To correct for multiple testing, the Benjamini–Hochberg procedure with a 10% false discovery rate was used. Furthermore, a supplementary analysis was executed to assess if the pooled logistic regression analysis was justified compared to a multilevel logistic regression model that corrects for between-study variation (by using study as a random effect). All analyses were performed using SAS (version 9.4).

Furthermore, we also checked data from the Dutch national AMR surveillance system, based on routine data from medical microbiological laboratories [17], to investigate if there was a seasonal trend in the monthly prevalence of ESBL-E/K BSIs (i.e. the proportion of BSIs caused by ESBL-E/K among all BSIs caused by *E. coli*/*K. pneumoniae*) from isolates taken between 2014 and 2016. ESBL production was estimated by ESBL confirmatory tests, or, when no data on confirmatory tests were available, by resistance against cefotaxime/ceftriaxone and/or ceftazidime.

A total of 8636 faecal samples of an equal amount of persons had been analysed in the three studies, and 5983 (69.3%) fulfilled the criteria to be included in this pooled analysis with a median age of 58 years (interquartile range 47–66) (Table 1). Of those, 260 tested positive for ESBL-E/K (4.3%; 95% CI 3.9–4.9%; range 2.6–7.4% per month). The month of sampling showed a statistically significant trend indicating that the observed prevalence differed between the months of the year (Mantel–Haenszel *χ*^2^ *P*-value of 0.036). In univariate analysis, monthly differences in the prevalence of ESBL-E/K carriage were observed in our data, with the highest prevalence in August and September compared to January (Table 2). In addition, travel to Africa, Asia or Latin America was statistically significant and associated with an increase in the ESBL-E/K prevalence (OR 2.85, 95% CI 2.10–3.86). The Benjamini–Hochberg procedure showed that travel to Africa, Asia or Latin America and September remained statistically significant. In multiple logistic regression analysis, the seasonal differences in the prevalence of ESBL-E/K carriage from the univariate analysis remained almost the same when results were adjusted for age, sex, previous antibiotic use and travel: compared to January, the odds of being an ESBL-E/K carrier was 1.88 in August (95% CI 1.02–3.49) and 2.25 in September (95% CI 1.30–3.90). When analyses were repeated without adjusting for antibiotic use during the previous 6 months (to increase the number of participants and ESBL-E/K carriers (*n* = 6395 participants of whom 291 (4.8%) were the carrier of ESBL-E/K)), results were very similar with a little increase in the ORs for August and September (data not shown). The random-effect estimates for the study were not statistically significant in the supplementary analysis, indicating that there was no significant variation between the studies. The ORs from the multilevel model were very similar to the ORs from the pooled logistic regression analysis.

**Table 2.** Descriptive characteristics, univariate and multiple logistic regression analysis of three pooled cross-sectional studies on the ESBL-E/K prevalence in the general Dutch population

Determinant	Total number (*n* = 5983)	Number of ESBL-E/K-positive (*n* = 260)	Prevalence	Univariate pooled odds ratio	Adjusted pooled odds ratio
	*n* (%)	*n* (%)	%	OR (95%CI)	OR (95%CI)
**Age group**					
18–<30 years	340 (5.7)	13 (5.0)	3.8		
30–<40 years	502 (8.4)	19 (7.3)	3.8		
40–<50 years	966 (16.1)	32 (12.2)	3.3		
50–<60 years	1490 (24.9)	70 (26.9)	4.7		
60–<70 years	1814 (30.3)	87 (33.2)	4.8		
70 years and older	871 (14.6)	39 (15.0)	4.5		
Age (per year increase)				1.01 (1.00–1.01)	1.01 (1.00–1.02)
**Sex**					
Male	2628 (43.9)	124 (47.3)	4.7	Ref.	Ref.
Female	3355 (56.1)	136 (52.3)	4.1	0.85 (0.67–1.09)	0.90 (0.69–1.16)
**Antibiotic use during last 6 months**					
No	5531 (92.5)	240 (92.3)	4.3	Ref.	Ref.
Yes	452 (7.6)	20 (7.7)	4.4	1.02 (0.64–1.63)	1.04 (0.65–1.66)
**Travel during last 6–12 months**^a^					
No travel or travel to Western/Northern Europe, North America, Australia or New Zealand	3742 (62.5)	131 (50.4)	3.5	Ref.	Ref.
Travel to Southern/Eastern Europe	1504 (25.1)	60 (23.1)	4.0	1.15 (0.84–1.56)	1.17 (0.86–1.60)
Travel to Africa, Asia or Latin America	737 (12.3)	69 (26.3)	9.4	2.85 (2.10–3.86)	2.95 (2.18–4.01)
**Month**					
January	603 (10.1)	21 (8.1)	3.5	Ref.	Ref.
February	486 (8.1)	20 (7.7)	4.1	1.19 (0.64–2.22)	1.12 (0.60–2.10)
March	540 (9.0)	14 (5.4)	2.6	0.74 (0.37–1.47)	0.70 (0.35–1.39)
April	513 (8.6)	21 (8.1)	4.1	1.18 (0.64–2.19)	1.17 (0.63–2.17)
May	532 (8.9)	21 (8.1)	4.0	1.14 (0.62–2.11)	1.14 (0.61–2.11)
June	602 (10.1)	23 (8.9)	3.8	1.10 (0.60–2.01)	1.07 (0.58–1.96)
July	203 (3.4)	10 (3.9)	4.9	1.44 (0.67–3.10)	1.42 (0.65–3.07)
August	343 (5.7)	22 (8.5)	6.4	1.90 (1.03–3.51)	1.88 (1.02–3.49)
September	512 (8.6)	38 (14.6)	7.4	2.22 (1.29–3.84)	2.25 (1.30–3.89)
October	599 (10.0)	28 (10.8)	4.7	1.36 (0.76–2.42)	1.29 (0.72–2.30)
November	672 (11.2)	24 (9.2)	3.6	1.03 (0.57–1.86)	1.04 (0.57–1.89)
December	378 (6.3)	18 (6.9)	4.8	1.39 (0.73–2.64)	1.34 (0.70–2.55)

a Depending on the study.

Finally, no clear seasonal trend could be observed in the monthly ESBL/E-K BSI prevalence data from the national antimicrobial resistance surveillance system: all Mantel–Haenszel *χ*^2^ *P*-values were non-significant, both overall and when a distinction was made between hospital-acquired (blood sample taken >2 days after admission) and community-acquired BSIs (data not shown).

In this pooled analysis with the data of three consecutive years (February 2014–April 2017) from 5983 individuals, ESBL-E/K carriage rates in the general population varied over the months of the year. Adjustment for age, sex and known risk factors for ESBL-E/K carriage (travel and antibiotic use) negligibly influenced the results from the univariate analysis and other factors seem to play a more important role to the exposure. We hypothesise that the higher ESBL-E/K carriage rate in August and September is caused by an increased chance of exposure to and/or acquisition of ESBL-E/K, potentially in combination with other factors including human behaviour/social and environmental factors.

As described above, increased incidence of enteric zoonotic diseases during warmer months including Salmonellosis, Campylobacteriosis and infections with Shiga toxin-producing *E. coli* has been reported in temperate regions [7]. This seasonality during warmer months also seems to be applicable to infections caused by *E. coli* and ESBL-E/K, while for non-ESBL-producing *K. pneumoniae*, conflicting results were found between different studies with regard to seasonal variations [10, 15]. Recent new insights into the distinction in seasonality between hospital-acquired and community-acquired BSIs, with only higher rates during warmer months for community-acquired BSIs and not for hospital-acquired infections [11, 12], suggest the existence of a certain mechanism for infection outside the hospital. Moreover, seasonality seems to be present in infections and carriage caused by ESBL-E as shown previously [14, 15]. Interestingly, the study by Gradel *et al*. on community-acquired bacteraemia caused by *E. coli* in Denmark observed peak dates for different subgroups in August and September [11], which are exactly the same months of increased ESBL-E/K carriage in the community in the present pooled analysis.

Several explanations for increased rates of infections and ESBL carriage of *E. coli* and *K. pneumoniae* during summer in humans have been suggested. However, the causal mechanisms driving this are generally unknown and different processes seem to counteract each other, like increased animal shedding *vs.* decreased survival outside the host with increasing temperatures. In addition, it is very likely that it is an interplay between environmental and human behavioural/social factors. The abundance and diversity of genes encoding ESBLs differ significantly between open community and livestock and their products, indicating that animal production and foodborne transmission have a lesser contribution to ESBL carriage in humans than previously assumed in the Netherlands [18], and human-to-human contact is the main driver for ESBL-E transmission [18–20]. Therefore, increased human–human exposure seems to be the most likely cause for the higher ESBL-E/K carriage rate in certain months. Increased transmission at population-level may consequently enable population-level selection of resistant strains [13]. Nevertheless, during warmer months, human exposure to contaminated food (i.e. undercooked meat) may be increased by different eating habits and diminished food hygiene (like during BBQs, picnics and eating more raw vegetables). Besides, increased exposure via environmental sources (like water and soil) and via animals could also play a role [13], potentially in combination with seasonal human behaviour such as performing more outdoor activities (i.e. beach and swimming in recreational waters), more frequent contact with family and friends, and increased sexual activity during summer [21]. However, the attribution of ESBL-carriage to environmental sources and contact with animals seems to be limited [20], which might explain why no effect of study (i.e. one of the studies was in a livestock-dense area) on the monthly variations was found in our analyses. In addition, seasonal changes in susceptibility to colonisation or infection are also possible. For example, there is an indication that photoperiod (day length)-driven physiologic changes are occurring in mammalian species, including some in humans. This possibly has an influence on the susceptibility of the host population for certain pathogens [22]. Furthermore, higher temperatures may facilitate the uptake of free genetic material or horizontal gene transfer, which is the case for plasmid-mediated ESBLs [13]. This increased gene transfer (in the host or the environment) might then be likely to facilitate population transmission [13]. Moreover, MacFadden *et al*. found that fluoroquinolones and *β*-lactams in general showed the strongest associations between temperature and resistance, which supports the mechanism-specific impacts of temperature on resistance [13]. Finally, Davenport *et al*. showed in a population with a minimal environmental and genetic variation that despite overall gut microbiome stability within individuals over time, there are consistent and significant population-wide shifts in microbiome composition across seasons. In general, a lower gut microbiome diversity was observed during summer than during winter [23]. This seasonal shift in microbiome composition might play a role in the chance of individuals to become colonised with ESBL (i.e. their susceptibility); however, at the moment this is still unclear.

The current analysis was performed by pooling data from three different cross-sectional studies in the Netherlands using univariate and multiple logistic regression analysis. All participants were from the general population, aged ≥18 years and similar laboratory techniques to identify ESBL-E/K carriers were performed. The supplementary analysis with random effects for the study did not alter our results. It was therefore decided to keep the analysis as simple as possible.

A limitation of the study is that we only had data available of reported travel during the last 6–12 months (depending on the study), and we did not know the exact timing of the reported travel relative to the faecal sampling. Therefore, we might not have been able to adjust our analyses completely for travel. A second limitation is that, although data were available for three consecutive years, the number of samples for some months in this period was too low to perform a longitudinal analysis check whether the effect observed in August and September occurred yearly.

Finally, the analysis of the national surveillance data showed no clear seasonal trend in the monthly proportion of BSIs caused by ESBL-E/K out of all *E. coli* and *K. pneumoniae* BSIs. A limitation of this analysis was, however, that the distinction between hospital-acquired and community-acquired infection based on the surveillance data might have been biased as the date of hospitalisation was missing in a substantial part of the infections. Furthermore, we could not include incidence data on ESBL-E/K BSIs because no monthly denominator is available in the surveillance system on routine diagnostics.

In conclusion, differences in monthly ESBL-E/K carriage rates in the general community were found in the Netherlands, which may indicate that the factors during warmer seasons could influence the probability of an individual to become colonised. However, no seasonal trend in the proportion of ESBL-E/K BSIs was seen in the national surveillance data, indicating that the increased ESBL-E/K carriage rate did not result in an increase in community-onset invasive infections. In addition to adjusting for known risk factors, we advise to consider seasonal fluctuations in carriage rates when designing future ESBL prevalence studies in the general community and among risk groups (such as persons in nursing homes, hospitals) in temporal regions, as these might partly adjust for human behavioural/social and environmental factors that affect ESBL-E/K carriage in the community. We therefore recommend to sample participants within the same time window (i.e. during the same season but preferably (the same fraction) year-round) and not consecutively.
